# Minimally Invasive Glaucoma Surgery: A Review of the Literature

**DOI:** 10.3390/vision7030054

**Published:** 2023-08-21

**Authors:** Michael Balas, David J. Mathew

**Affiliations:** 1 Temerty Faculty of Medicine, University of Toronto, Toronto, ON M5S 1A8, Canada; michael.balas@mail.utoronto.ca; 2 Donald K. Johnson Eye Institute, Krembil Research Institute, University Health Network, Toronto, ON M5T 0S8, Canada; 3 Department of Ophthalmology and Vision Sciences, University of Toronto, Toronto, ON M5T 2S8, Canada; 4 Department of Laboratory Medicine and Pathobiology, University of Toronto, Toronto, ON M5S 1A8, Canada

**Keywords:** minimally invasive glaucoma surgery, MIGS, glaucoma, intraocular pressure, trabecular outflow, suprachoroidal outflow, conjunctival bleb-forming procedures, surgical advances, literature review

## Abstract

Minimally invasive glaucoma surgery (MIGS) has emerged as a novel approach in the glaucoma treatment spectrum, offering a range of diverse procedures and devices aimed at reducing intraocular pressure (IOP). MIGS can be broadly classified into several categories: those that enhance trabecular outflow (Trabectome, iStent, Hydrus Microstent, Kahook Dual Blade, high frequency deep sclerotomy, and gonioscopy-assisted transluminal trabeculotomy), those that augment suprachoroidal outflow (CyPass Microstent and iStent Supra), those that target Schlemm’s canal (TRAB360 and the OMNI Surgical System, Streamline, and Ab Interno Canaloplasty), and conjunctival bleb-forming procedures (EX-PRESS Glaucoma Filtration Device, Xen Gel Stent and PreserFlo MicroShunt). MIGS is considered to have a shorter surgical time and fewer severe complications when compared to traditional glaucoma surgeries such as trabeculectomy and glaucoma drainage device implantation (Ahmed, Baerveldt, and Molteno valves). This literature review comprehensively examines the distinct MIGS devices and procedures, their underlying mechanisms, and clinical outcomes, emphasizing the importance of evaluating the efficacy and complications of each approach individually. As the field of MIGS continues to evolve, it is crucial to prioritize high-quality, long-term studies to better understand the safety and effectiveness of these innovative interventions in glaucoma management.

## 1. Introduction

Minimally invasive glaucoma surgery (MIGS) has emerged in the past two decades as a promising approach to address many unmet needs in glaucoma management [1,2,3]. MIGS encompasses a broad range of surgical techniques and devices which aim to lower IOP with a more favorable safety profile compared to traditional glaucoma surgeries [4]. These procedures are typically characterized by their ab interno approach, rapid recovery time, and preservation of the conjunctiva for potential future glaucoma surgeries. MIGS has been designed to target different aqueous humor outflow pathways, including Schlemm’s canal, the suprachoroidal space, and the subconjunctival space.

### 1.1. Terminology

As the field of MIGS continues to evolve, it is essential to understand the terminology associated with these procedures and devices to facilitate clear communication among researchers, clinicians, and patients. MIGS can be broadly classified based on itstarget anatomical site, approach, and mechanism of action [5].

The term “ab interno” refers to MIGS procedures that are performed through an internal approach, typically via a clear corneal incision. In contrast, “ab externo” procedures involve an external approach, usually requiring a scleral or conjunctival incision. Ab interno MIGS is generally considered less invasive and have a more favorable safety profile due to their conjunctiva-sparing nature, which reduces the risk of complications such as infection, scarring, and hypotony [2,6].

MIGS devices can be categorized based on the primary anatomical site they target to enhance aqueous humor outflow and reduce intraocular pressure (IOP). These categories include trabecular meshwork bypass, supraciliary shunts, and subconjunctival filtration devices. Trabecular meshwork bypass devices, such as iStent and Hydrus Microstent, aim to improve the outflow of aqueous humor by bypassing the trabecular meshwork, the primary site of resistance in the conventional outflow pathway. Suprachoroidal shunts, like CyPass Micro-Stent and iStent Supra, target the supraciliary space, creating an alternative pathway for aqueous humor to exit the eye. Subconjunctival filtration devices, including XEN Gel Stent and PreserFlo MicroShunt, facilitate the creation of a drainage pathway from the anterior chamber to the subconjunctival space, allowing aqueous humor to exit the eye and be absorbed by the conjunctiva and episcleral vasculature [1].

### 1.2. Advantages and Limitations

The benefits and drawbacks of MIGS are often evaluated in comparison to traditional glaucoma surgeries, such as trabeculectomy and glaucoma drainage devices. These advantages and limitations play a crucial role in determining the position of MIGS within the glaucoma treatment paradigm.

Initial management of glaucoma typically involves pharmacotherapy and laser therapy, which are associated with fewer risks compared to traditional glaucoma surgeries [4]. Conventional glaucoma surgeries carry the potential for vision-threatening intraoperative and postoperative complications, including hypotony, infection, suprachoroidal hemorrhage, cataract formation, and the need for additional surgeries [7]. The Primary Tube Versus Trabeculectomy study reported postoperative complications in 29% and 41% of patients in the tube and trabeculectomy groups, respectively, after one year of follow-up. Serious complications that led to the loss of two or more Snellen lines or required repeat surgery occurred in 1% and 7% of patients, respectively [8]. In terms of complication risk, MIGS occupies an intermediate position between pharmacotherapy and laser therapy, which have lower risks, and traditional glaucoma surgeries, which have higher risks [4,9].

MIGS offers several advantages compared to traditional glaucoma surgeries, including a better safety profile and faster recovery time. It is generally indicated for the treatment of mild to moderate glaucoma, as the IOP-lowering effect of MIGS is less pronounced than that of traditional glaucoma surgeries [1,2,3,6]. However, the limitations of MIGS should also be considered. These procedures may not achieve the same degree of IOP reduction as traditional surgeries, potentially limiting their efficacy in cases of advanced glaucoma or in patients with a low target IOP.

Moreover, while MIGS has demonstrated promising results in the short to medium term, long-term outcomes and comparative effectiveness among different MIGS techniques are still under investigation [10]. The cost-effectiveness of MIGS compared to traditional glaucoma surgeries has also yet to be definitively established [11].

In summary, MIGS offers several advantages over traditional glaucoma surgeries, such as an improved safety profile and faster recovery. However, limitations, including potentially inferior IOP-lowering effects and uncertainty regarding long-term outcomes and cost-effectiveness, should be carefully considered when determining the role of MIGS in the glaucoma treatment paradigm.

The primary objective of this literature review is to provide an up-to-date, comprehensive summary of the current evidence on MIGS, focusing on their safety, efficacy, and the specific patient populations that may benefit the most from these procedures. We will explore the various MIGS devices and techniques, including trabecular micro-bypass stents, Schlemm’s canal scaffolding, suprachoroidal shunts, and subconjunctival filtration devices. Furthermore, we will discuss the advantages and limitations of each MIGS approach, the impact of MIGS on the glaucoma treatment paradigm, and the future directions of research in this rapidly evolving field. Given the rapidly evolving nature of this field and the growing number of approved MIGS devices, it is crucial to regularly assess and synthesize the available evidence to guide clinicians, researchers, and healthcare policymakers in making informed decisions about the use of MIGS in glaucoma treatment.

## 2. Literature Search Details

A comprehensive literature search was conducted using the PubMed database to identify relevant MIGS studies published in English. The search strategy involved the utilization of the following keywords and terms: *Trabectome*, *iStent*, *Hydrus*, *trabecular stent*, *sclerotomy*, *gonioscopy-assisted transluminal trabeculotomy*, *Kahook Dual Blade*, *Schlemm’s canal cannulation*, *Schlemm’s canal dilation*, *Ab Interno Canaloplasty*, *CyPass*, *suprachoroidal shunt*, *suprachoroidal stent*, *EX-PRESS*, *XEN*, *gel stent*, *InnFocus*, *and poly(styrene-block-isobutylene-block-styrene)*. To enhance the search sensitivity and ensure the inclusion of all relevant publications, both Medical Subject Headings (MeSH) terms and free-text keywords were used in various combinations.

In addition to the primary search, the reference lists of the identified MIGS studies were thoroughly examined to uncover any additional eligible studies that might have been missed during the initial search. The search period was set from 1 January 2000 to 1 March 2023 to capture the most up-to-date evidence in the rapidly evolving field of MIGS. Conference abstracts were not included in the search to maintain a focus on peer-reviewed, full-length publications that provide more extensive data and methodological details.

### Indications and Contraindications

MIGS is generally indicated for the treatment of mild to moderate open-angle glaucoma (OAG) in patients who have failed to achieve adequate IOP control through conservative management approaches, such as pharmacotherapy and laser therapy [1,2,3,6]. These procedures may also be considered for patients with uncontrolled IOP who are intolerant or non-adherent to their glaucoma medications due to side effects or complex dosing regimens. In some cases, MIGS may be performed in conjunction with cataract surgery, offering a combined approach to address both glaucoma and cataracts in a single surgical intervention. This approach can streamline patient care, reduce the overall number of surgeries, and potentially improve patient satisfaction and quality of life [12,13].

The contraindications for MIGS are largely dependent on the specific procedure or device being utilized and the patient’s clinical characteristics [14]. However, some general contraindications for MIGS include advanced glaucoma with severe visual field loss, uncontrolled uveitic glaucoma, neovascular glaucoma, and angle-closure glaucoma (ACG) without prior laser peripheral iridotomy (LPI) or in cases where the angle remains closed after LPI. MIGS targeting the trabecular meshwork, such as iStent and Trabectome, may be contraindicated in patients with significant peripheral anterior synechiae or an occludable angle due to the potential risk of angle closure or progressive synechial closure postoperatively. Similarly, MIGS targeting the supraciliary or suprachoroidal space, such as CyPass and iStent Supra, may be contraindicated in patients with a history of uveitis, scleritis, or other inflammatory ocular conditions that could compromise the surgical outcome.

## 3. Devices, Procedures, and Surgical Techniques

**Figure 1:**
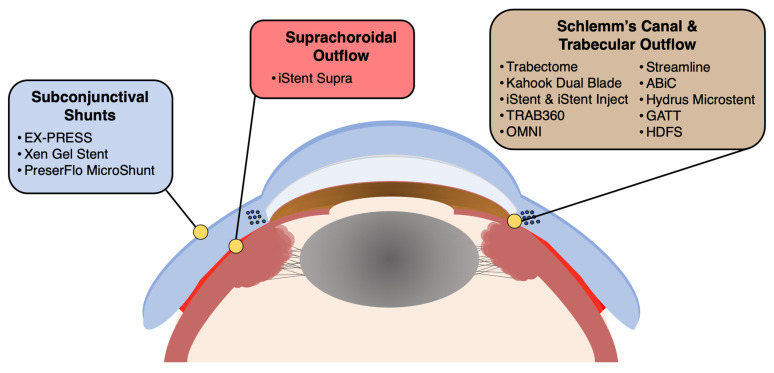
Illustration depicting the coronal cross-section of the anterior segment of the eye, highlighting the anatomic localization of each minimally invasive glaucoma surgery (MIGS) device category, including subconjunctival shunts, suprachoroidal outflow, as well as Schlemm’s canal targeted and trabecular outflow devices. ABiC: Ab Interno Canaloplasty; GATT: gonioscopy-assisted transluminal trabeculotomy; HDFS: high-frequency deep sclerotomy.

In the realm of glaucoma management, a diverse array of devices, procedures, and surgical techniques have emerged to address the need for effective IOP reduction and preservation of visual function. This section provides an overview of some of the most promising and widely used approaches in the field of surgical glaucoma treatment, offering a deeper understanding of their mechanisms, benefits, and potential applications for various patient populations (Table 1). Figure 1 highlights the anatomical location of each device and device category.

### 3.1. Trabectome and Kahook Dual Blade

The Trabectome, developed by NeoMedix Corporation, and the Kahook Dual Blade (KDB), developed by New World Medical, have emerged as valuable tools in the management of glaucoma, specifically targeting the trabecular meshwork to improve aqueous humor outflow and consequently reduce IOP [15,16,17]. Although both devices share the same goal of enhancing outflow, they employ distinct mechanisms and techniques to achieve this outcome.

The Trabectome, a microelectrocautery device, utilizes high-frequency electrical energy to ablate and remove a strip of the trabecular meshwork and the inner wall of Schlemm’s canal, thereby facilitating aqueous humor outflow directly into the canal [15,18]. This approach minimizes tissue trauma, leading to reduced inflammation and scarring postoperatively. However, compared to other approaches, there is an increased risk of intraoperative blood reflux, which occurs due to the high-frequency ablation and direct communication with the blood-filled Schlemm’s canal. While this reflux often clears spontaneously and does not necessarily affect the postoperative outcome, it may increase the complexity of the intraoperative visual field, potentially influencing surgical ease and duration [19]. Despite this, studies have reported favorable outcomes with the Trabectome, demonstrating significant IOP reduction and a decrease in the number of glaucoma medications required by patients [15]. Approved by the FDA in 2004, the Trabectome continues to offer a promising approach for facilitating enhanced aqueous humor outflow in glaucoma patients while simultaneously striving to minimize tissue damage and subsequent inflammatory responses.

The Kahook Dual Blade (KDB), approved in 2015, features a novel dual-sided, pointed tip that enables precise incisions to create a trabecular meshwork window. This surgical instrument lifts and excises a strip of the trabecular meshwork, resulting in an unobstructed flow of aqueous humor into Schlemm’s canal [20]. The KDB’s unique design allows for minimal collateral damage to surrounding tissues, contributing to faster recovery times and fewer complications. Clinical studies have shown that the KDB not only reduces IOP effectively but also maintains this reduction over time [16,21]. Moreover, the KDB has been found to be safe, with minimal adverse events such as transient hyphema or IOP spikes [22].

Both the Trabectome and KDB can be utilized as standalone procedures or in conjunction with phacoemulsification cataract surgery, providing a versatile treatment strategy for patients with mild to moderate glaucoma [23]. Combining these MIGS techniques with cataract surgery has been reported to yield even greater IOP reduction and a decrease in glaucoma medication dependence, further solidifying the role of these devices in comprehensive glaucoma management [24,25].

### 3.2. iStent and iStent Inject

The iStent and iStent Inject, developed by Glaukos Corporation, are MIGS devices designed to enhance aqueous humor outflow and reduce IOP in patients with mild to moderate open-angle glaucoma by bypassing the trabecular meshwork [26,27]. These heparin-coated, L-shaped titanium stents are biocompatible and exhibit excellent long-term stability, minimizing the risk of complications and enhancing patient outcomes.

The iStent, approved by the FDA in 2012, marked the introduction of the first trabecular micro-bypass stent. Implanted through a clear corneal incision during a minimally invasive procedure, the iStent is inserted into Schlemm’s canal to bypass the trabecular meshwork and directly enhance aqueous humor outflow [26]. Studies have shown that the iStent, whether combined with cataract surgery or not, results in a significant reduction in IOP and the need for ocular hypotensive medications [28,29].

In response to the success of the iStent, the iStent Inject was developed and approved by the FDA in 2018. This device features a preloaded injector system that allows for the implantation of two stents, providing an increased surface area for aqueous humor outflow and further enhancing IOP reduction [27]. The iStent Inject, like its predecessor, can be performed as a standalone procedure or in conjunction with cataract surgery. Various studies have shown that the iStent Inject is preferred in terms of safety and efficacy overall, especially with respect to the benefits of the dual-stent approach [27,30].

Compared to other techniques, both the iStent and iStent Inject have the potential difficulty in implantation due to the small size of the devices. Misplacement or malposition of the stents can lead to suboptimal IOP control [31]. This necessitates careful and precise surgical technique and experience to ensure correct placement within Schlemm’s canal for optimal performance and patient outcomes. Despite this challenge, when implanted correctly, these devices have proven to be effective and safe options in the management of mild to moderate open-angle glaucoma.

### 3.3. High-Frequency Deep Sclerotomy

High-frequency deep sclerotomy (HFDS), originally known as sclerothalamotomy ab interno (STT ab interno), aims to reduce IOP by creating microincisions in the sclera using high-frequency electric current, thereby enhancing uveoscleral outflow [32,33]. The HFDS procedure involves the placement of a specialized diathermic probe (Oertli Instrumente) equipped with a high-frequency generator into the suprachoroidal space, creating multiple small incisions in the sclera. These microincisions facilitate the flow of aqueous humor from the anterior chamber to the suprachoroidal space, thereby bypassing the conventional outflow pathways and effectively lowering IOP. The current delivered by the HFDS probe creates microperforations in the sclera without causing any collateral damage to the surrounding tissues, ensuring a minimally invasive and tissue-sparing approach [33,34].

Initial studies investigating the use of HFDS in glaucoma management have shown encouraging results, with significant reductions in IOP and a favorable safety profile [32,33,35]. In addition, HDFS can be combined with cataract surgery while avoiding the risk of implant-related complications and has a relatively short learning curve for surgeons already experienced in glaucoma surgery. However, despite the promising initial results, further large-scale, randomized, controlled trials are necessary to evaluate the long-term safety and efficacy of HFDS. Future research should also focus on identifying the optimal patient population for HFDS and on comparing the performance of HFDS with other established MIGS procedures.

### 3.4. TRAB360, OMNI, Streamline, and ABiC

Schlemm’s canal targeted procedures have emerged as another category of MIGS, aiming to improve trabecular outflow and decrease IOP by directly targeting and enhancing the function of Schlemm’s canal. These procedures include the TRAB360, OMNI, Streamline, and Ab Interno Canaloplasty (ABiC).

The TRAB360 (Sight Sciences) is an advanced trabeculotomy procedure that offers a minimally invasive approach to enhance aqueous humor outflow by directly targeting Schlemm’s canal. Utilizing a flexible microcatheter, the TRAB360 cannulates and viscodilates Schlemm’s canal, subsequently bypassing the trabecular meshwork. The device, inserted through a clear corneal incision, is carefully advanced to create a 360-degree trabeculotomy. FDA-approved in 2013, TRAB360 has demonstrated promising results in both adult and pediatric OAG by lowering IOP and reducing the need for additional glaucoma medications [36,37]. Its unique design features a highly flexible and maneuverable catheter that enables precise control and access to Schlemm’s canal, translating to reduced surgical time and faster patient recovery. However, the TRAB360 procedure is associated with an increased risk of hyphema due to the circumferential trabeculotomy [36]. This risk arises from the disruption of collector channels and the potential for blood reflux into the anterior chamber during the procedure. While generally self-limiting, this hyphema may contribute to transient IOP spikes in the immediate postoperative period. Despite this, the TRAB360 has been a valuable tool in the management of glaucoma and has since been replaced in the market by its cousin, the OMNI Surgical System (Sight Sciences).

The OMNI Surgical System (Sight Sciences) is a comprehensive two-step MIGS procedure that effectively combines viscodilation (VISCO360) of Schlemm’s canal and trabeculotomy (TRAB360) to enhance aqueous humor outflow and lower intraocular pressure. The first step involves utilizing a specialized microcatheter to deliver a viscoelastic material into Schlemm’s canal, effectively dilating the canal and the collector channels. Following the viscodilation process, the OMNI device performs a 360-degree trabeculotomy, further enhancing aqueous humor outflow. This integrated handheld system offers a solution that addresses multiple points of outflow resistance in the conventional outflow pathway, resulting in improved surgical outcomes and better management of intraocular pressure. FDA-approved in 2017, the OMNI Surgical System has demonstrated its effectiveness in reducing IOP and minimizing the reliance on glaucoma medications in patients with mild-to-moderate OAG, along with a favorable safety profile and low incidence of complications [38,39].

Streamline (New World Medical) is a MIGS procedure designed to enhance aqueous humor outflow by targeting Schlemm’s canal through canaloplasty, which involves the cannulation and viscodilation of Schlemm’s canal without performing a trabeculotomy. The device consists of a single-use and disposable stainless-steel cutting inner cannula with a polymer outer sleeve, inserted via a clear corneal incision. As the outer cannula retracts, it creates a 150 μm diameter goniotomy while simultaneously delivering approximately 7 μL of viscoelastic material into Schlemm’s canal, effectively dilating the canal and connected collector channels. This dilation facilitates improved aqueous humor outflow and helps lower IOP in patients with glaucoma. The Streamline device, composed of biocompatible materials, ensures optimal safety and performance during the procedure and allows for extended goniotomies over multiple clock hours as needed. It received FDA approval in 2021, with various clinical trials underway. An interim analysis of a prospective non-randomized trial revealed a significant reduction in mean IOP and number of medications at the 6-month follow-up [40]. It is suitable for both phakic and pseudophakic eyes. Further research is warranted to evaluate the long-term efficacy, safety, and potential complications associated with the Streamline Surgical System, as well as to compare its outcomes to other MIGS procedures.

Ab Interno Canaloplasty (ABiC, Ellex iScience) aims to enhance aqueous humor outflow and reduce IOP by targeting and viscodilating Schlemm’s canal, the trabecular meshwork, and the distal outflow system, thereby restoring the natural outflow pathways. The ABiC procedure employs a flexible 250 µm fiber optic microcatheter, the iTrack microcatheter system (Ellex iScience), inserted through a clear corneal incision to cannulate dilate the Schlemm’s canal and distal outflow channels with viscoelastic material. This results in improved IOP control with minimal tissue disruption, showing promise as an effective treatment option for mild-to-moderate primary OAG that is relatively low-risk and easy to use [41]. Numerous early studies have shown favorable reductions in IOP and medications, although additional, large-scale, and long-term research will be necessary to further characterize its efficacy and risk of adverse events [42,43].

### 3.5. Hydrus Microstent

The Hydrus Microstent, developed by Ivantis Inc., is a MIGS device designed to improve aqueous humor outflow and reduce IOP by dilating Schlemm’s canal and bypassing the trabecular meshwork [44]. Approved by the FDA in 2018, the Hydrus Microstent is a flexible, biocompatible nitinol stent that spans approximately 90 degrees of the canal. Implantation of the Hydrus Microstent is performed through a clear corneal incision, typically in conjunction with cataract surgery, which allows for a more efficient and streamlined surgical experience. The device is inserted into Schlemm’s canal using a preloaded, specially designed injector, ensuring precise and accurate placement [45]. However, there still exists the potential for malposition or migration of the stent, which can occur due to incorrect placement or unexpected postoperative shifts [44]. While a careful surgical technique can mitigate this risk, it nevertheless represents a specific consideration for surgeons employing this device.

The efficacy and safety of the Hydrus Microstent have been evaluated through numerous clinical trials, including the HORIZON study, a multicenter, randomized controlled trial comparing the outcomes of combined cataract surgery with Hydrus Microstent implantation versus cataract surgery alone [46,47]. The results of the HORIZON study demonstrated that patients in the Hydrus Microstent group achieved durable IOP-lowering effects and reduced medication burden, in addition to lowering the risk of future glaucoma surgeries without increasing the risk of adverse events [46,47].

While the Hydrus Microstent has shown promising results in terms of IOP reduction and safety profile, it is important to acknowledge the need for long-term follow-up studies to assess the durability of its effects. At five years, however, the implant appears to maintain its IOP-lowering effects without adversely affecting the corneal endothelium or resulting in other complications [46]. These findings are further supported by observational studies as well [48,49]. Furthermore, the optimal patient population and the role of the Hydrus Microstent in various glaucoma subtypes require further investigation [45,50].

### 3.6. Gonioscopy-Assisted Transluminal Trabeculotomy (GATT)

GATT is a MIGS technique that improves aqueous humor outflow by creating a 360-degree or smaller trabeculotomy through an ab interno approach [51]. This procedure offers a unique advantage over other MIGS techniques, as it does not rely on a specific device or manufacturer. Instead, GATT utilizes a microcatheter and a flexible suture, such as a 5-0 or 6-0 polypropylene monofilament, to facilitate the circumferential unroofing of Schlemm’s canal. The GATT procedure is initiated by creating a clear corneal incision, followed by the introduction of a microcatheter into the anterior chamber. The microcatheter is then inserted into Schlemm’s canal under gonioscopic guidance and carefully threaded circumferentially around the canal. Once the microcatheter tip is visualized in the opposite side of the anterior chamber angle, the suture is tied to the microcatheter and subsequently drawn through the entire length of Schlemm’s canal. The suture is then gently pulled, effectively unroofing the trabecular meshwork and creating up to a 360-degree trabeculotomy [51].

GATT has been shown to be a versatile and effective option for managing mild to moderate open-angle glaucoma, including primary OAG, juvenile OAG, and pseudoexfoliative glaucoma [52]. The procedure can be performed as a standalone intervention or combined with cataract surgery, allowing for a more comprehensive and individualized treatment strategy. Studies have demonstrated significant IOP reduction and reduced reliance on anti-glaucoma medications following GATT, with a favorable safety profile and minimal adverse events such as transient hyphema and IOP spikes [53,54]. While GATT is effective and adaptable, it carries unique complications such as an increased risk of hyphema from the extensive unroofing of Schlemm’s canal [55]. Typically, these transient events resolve spontaneously or with conservative management, and postoperative IOP spikes are generally well controlled with medication.

Recent advancements in the GATT technique involve modifications to further enhance the procedure’s safety and efficacy. One such innovation is the development of microsurgical instruments specifically designed for GATT, such as illuminated or thermally marked microcatheters, which have the potential to improve procedural outcomes and ease the learning curve for surgeons [56].

### 3.7. iStent Supra and CyPass

The iStent Suprachoroidal Bypass System (iStent Supra) is a MIGS device engineered to harness the suprachoroidal outflow pathway for lowering IOP. This device utilizes the eye’s natural suprachoroidal space as a conduit for aqueous humor drainage, bypassing the conventional trabecular meshwork, which is often the site of increased resistance in glaucomatous eyes. This particular outflow route takes advantage of the pressure gradient between the anterior chamber and the suprachoroidal space, ensuring effective drainage and consequent IOP reduction.

The iStent Supra, developed by Glaukos Corporation, is constructed from biocompatible polyethersulfone (PES) material with a porous titanium coating. It is implanted ab interno through a clear corneal incision and facilitates aqueous humor drainage into the suprachoroidal space. The device’s design and material choice aim to improve biocompatibility, reduce fibrosis, and promote stable implantation [57]. The iStent Supra has received CE Mark approval in Europe and has shown significant IOP reductions in the majority of patients with OAG [58,59]. Due to its position in the suprachoroidal space, a unique complication could arise in the form of implant obstruction. The suprachoroidal space is a potential space with variable thickness that can be affected by changes in choroidal volume or IOP, which might, in some cases, lead to obstruction of the iStent Supra [60]. This potential risk should be considered during patient selection, and meticulous surgical technique should be employed to ensure accurate placement of the device. Ongoing clinical trials in the US are underway to further evaluate the device’s efficacy, safety, and potential role in glaucoma management.

Previously, another device, the CyPass, used a similar approach. Developed by Alcon Laboratories, the CyPass was approved by the FDA in 2016 after demonstrating promising IOP reduction and safety profile in initial studies [60,61]. It utilized a polyimide material for its construction. This flexible, tube-like device is designed to be implanted ab interno through a clear corneal incision and placed into the supraciliary space, creating a direct conduit for aqueous humor to bypass the trabecular meshwork and flow into the suprachoroidal space. However, in 2018, the COMPASS-XT study revealed a higher rate of corneal endothelial cell loss in patients with the CyPass compared to those who underwent cataract surgery alone [62,63]. Consequently, Alcon Laboratories voluntarily withdrew the device from the market to further investigate these concerns and refine the device’s design to mitigate potential long-term risks.

The incident involving CyPass emphasizes the critical role that long-term safety and efficacy data play in the development, approval, and post-market surveillance of MIGS devices. It is a reminder that while initial studies can show promise, a complete and accurate understanding of a device’s safety profile and therapeutic potential only emerges over time and with large-scale, diverse use. It also serves as a clear example of the impact that robust post-market surveillance and continuous reassessment can have on patient safety. Alcon’s decision to voluntarily withdraw CyPass from the market in light of new safety concerns sets a precedent for responsiveness and responsibility, which should be the standard across the medical device industry. The comparison between the CyPass and the iStent Supra further highlights the importance of device-specific design and material properties in influencing therapeutic performance and safety profiles. It underscores the need for individualized assessment of each MIGS device and an understanding that while they may share similar mechanisms of action, their distinct attributes can result in differing patient outcomes.

### 3.8. EX-PRESS Glaucoma Filtration Device, Xen Gel Stent, and PreserFlo MicroShunt

The EX-PRESS Glaucoma Filtration Device, Xen Gel Stent, and the PreserFlo MicroShunt (previously InnFocus MicroShunt) are all minimally invasive devices designed to create subconjunctival drainage pathways for aqueous humor, effectively reducing IOP in patients with glaucoma [64,65].

The EX-PRESS Glaucoma Filtration Device, developed by Alcon, has been on the market since 2002 and offers a reliable and less invasive alternative to trabeculectomy for the treatment of glaucoma. This device consists of a stainless-steel tube measuring 2.64 mm in length and 400 microns in external diameter [65,66]. There are two models, the P-50 (50 micron lumen) and the P-200 (200 micron lumen). The smaller lumen of the P-50 is designed to provide a more controlled, slower rate of aqueous humor outflow, which may reduce the risk of hypotony. The EX-PRESS device is inserted under a partial-thickness scleral flap, much like in traditional trabeculectomy, to divert aqueous humor from the anterior chamber into the subconjunctival space. After performing a corneal paracentesis, a small partial-thickness scleral flap is created, and the device is inserted into the anterior chamber through the sclera, creating a fistula that allows the aqueous humor to bypass the trabecular meshwork. The scleral flap is then closed with sutures, which can be titrated postoperatively to control the flow of fluid and maintain an optimal IOP [65,66].

Studies have shown that the EX-PRESS device provides comparable IOP reduction to trabeculectomy but with fewer complications and a more predictable postoperative course [67,68,69,70]. Specifically, the EX-PRESS device has been associated with less hypotony, choroidal effusion, and hyphema, as well as faster visual recovery. However, it is important to note that long-term success with the EX-PRESS device still requires careful postoperative management, including frequent follow-up visits and potential suture adjustments. Moreover, compared to other devices, its larger size may lead to potential issues with device malposition or extrusion. In cases of malposition, the tube may not correctly divert aqueous humor, which could lead to suboptimal IOP control [71]. In rare instances, the device can extrude, which necessitates additional surgical intervention [72,73,74]. Proper surgical technique and postoperative management can greatly reduce the likelihood of these complications [75].

The Xen Gel Stent, manufactured by Allergan, is composed of a hydrophilic, porcine-derived gelatin material that is biocompatible and flexible, with a length of 6 mm and diameters of 45, 63, and 140 microns (Xen 45 is the only version that is currently commercially available) [76]. The ab interno procedure involves inserting the Xen Gel Stent through a clear corneal incision, positioning it within the trabecular meshwork and extending it into the subconjunctival space. By creating a new drainage pathway that bypasses the trabecular meshwork, the Xen implant facilitates aqueous humor outflow from the anterior chamber to the subconjunctival space, where it is absorbed by the surrounding tissue [76,77]. Approved by the FDA in 2016, the Xen Gel Stent is well suited for patients with moderate to severe glaucoma who may require greater IOP reduction than other MIGS procedures can provide. It can be performed as a standalone procedure or in combination with cataract surgery. Studies with the currently available Xen 45 have shown that it leads to significant reductions in IOP and medication use, both as a solo procedure or in combination with cataract surgery [78,79,80,81,82].

Intraoperatively, issues such as bleeding or the need for device repositioning may occur. Postoperative complications can include transient hypotony, temporary IOP spikes, or, more rarely, severe outcomes such as endophthalmitis, blebitis, or avascular filtering bleb [76,83,84]. In a minority of cases, complications such as implant migration, stent–iris touch, endothelial cell loss, and late wound leak have been reported [76,85,86,87]. Furthermore, postoperative needling might be required in a significant number of cases, with rates ranging from 30 to 50% [76,88,89]. By understanding these potential risks, clinicians can better tailor the management strategies, optimizing the beneficial impact of the Xen Gel Stent in glaucoma treatment.

The PreserFlo MicroShunt, developed by Santen Pharmaceutical Co., Ltd., is a small, flexible tube made of a biocompatible material known as poly(styrene-block-isobutylene-block-styrene), or SIBS, which is designed to minimize inflammation, scarring, and fibrosis [90]. Measuring 8.5 mm in length with a 70 µm lumen and 350 µm outer diameter, the PreserFlo MicroShunt works by diverting aqueous humor from the anterior chamber to the subconjunctival space, creating a filtration bleb and effectively lowering IOP. The ab externo surgical approach involves a small conjunctival incision and a scleral flap to create a pocket for the device. Once implanted, the device’s distal end is tucked under the conjunctiva and Tenon’s capsule, allowing aqueous humor to flow into the subconjunctival space [90,91]. It also features a 1.1 mm wide fin located 4.5 mm from the anterior tip, ensuring the device remains positioned appropriately and in the proper orientation within the anterior chamber. The PreserFlo MicroShunt received CE Mark approval in Europe in 2012 and was approved by the US FDA in 2021, expanding the array of MIGS options available for glaucoma management. Similar to the Xel Gel Stent, the PreserFlo MicroShunt is particularly suited for patients with moderate to advanced glaucoma who require a more significant IOP reduction than can be achieved with other MIGS procedures. Multiple studies on the PreserFlo Ab-Externo MicroShunt demonstrate its effectiveness in reducing IOP and medication usage in patients with primary OAG [91]. Surgical success rates have ranged from 70 to 95% at 1 to 3 years postoperatively, with transient complications such as hypotony and choroidal detachments being reported but generally resolving spontaneously [92,93,94,95]. Lower doses of mitomycin-C (MMC) and a diagnosis of secondary OAG have been associated with higher surgical failure rates.

A comparative study between the Xen and MicroShunt implants augmented with MMC in patients with primary OAG found that both implants effectively lowered IOP and reduced the need for postoperative IOP-lowering medications [96]. After 24 months of follow-up, qualified success rates were 73% for Xen implantation and 79% for MicroShunt implantation, with mean IOP dropping to the low teens in both groups. The incidence of hypotony and hypotony-related complications was low in both groups [96]. Another study using anterior segment optical coherence tomography (AS-OCT) revealed that XEN implantation resulted in a higher proportion of subconjunctival separation type blebs initially, which transitioned to a uniform pattern over time, while the PreserFlo MicroShunt had a higher prevalence of multiple internal layer blebs, which became microcystic multiform blebs over time [97]. These differences in bleb morphology may have implications for the long-term success and need for revisions. Further research is needed to determine the optimal concentration of MMC and to establish the long-term effects of the implant materials, in addition to investigating the clinical significance of these variations in bleb characteristics.

The EX-PRESS Glaucoma Filtration Device, Xen Gel Stent, and PreserFlo MicroShunt all represent subconjunctival shunts that create a new outflow pathway for aqueous humor, hence decreasing IOP. However, their design, implantation procedure, and resulting bleb morphology are different. The choice between these devices should be guided by individual patient needs and the surgeon’s familiarity with the device and technique. Patients with moderate to advanced glaucoma who have failed medical or laser treatment are generally considered for these procedures, with particular consideration for the risk-benefit ratio of each device. The EX-PRESS device may be a good option for patients with a higher risk of hypotony or who require a rapid recovery of visual acuity. The Xen Gel Stent may be considered in combination with cataract surgery or for patients with less severe glaucoma. The PreserFlo MicroShunt may be favored in patients who are at high risk for filtration surgery failure or who have shown intolerance to anti-metabolites.

As the landscape of glaucoma treatment continues to evolve, a thoughtful and patient-centered approach will remain at the heart of successful disease management. This ongoing journey toward refining our surgical armamentarium not only emphasizes the importance of continued innovation in glaucoma care but also underscores the need for robust, long-term clinical trials to inform future practice.

## 4. Postoperative Course and Outcomes

MIGS procedures generally have a favorable postoperative course, with most patients experiencing rapid recovery and minimal discomfort compared to traditional glaucoma surgeries [1,2,6]. The minimally invasive nature of these procedures, combined with the preservation of conjunctival and scleral tissue, contributes to a reduced risk of complications and a shorter healing period.

Following MIGS, patients can usually resume their normal activities within a few days to a week, although they may be advised to avoid strenuous activities, swimming, or exposure to dust and debris for a short period. Postoperative care typically includes a regimen of topical antibiotics and anti-inflammatory medications, as well as regular follow-up visits to monitor IOP and assess the surgical outcome [98].

The primary outcome of MIGS is the reduction in IOP, which is essential for halting or slowing the progression of glaucomatous optic neuropathy. While MIGS has demonstrated moderate IOP-lowering efficacy, it may not achieve the same magnitude of IOP reduction as traditional glaucoma surgeries, such as trabeculectomy and glaucoma drainage devices [1,2,6]. However, the IOP reduction achieved with MIGS is often sufficient for the management of mild to moderate OAG [11,60].

Another critical outcome of MIGS is its safety profile. MIGS is generally associated with fewer complications and a lower risk of vision-threatening events compared to traditional glaucoma surgeries [1,2,6]. Common complications of MIGS include transient hyphema, IOP spikes, and device-related issues, such as malposition or migration. Serious complications, such as hypotony, infection, or suprachoroidal hemorrhage, are relatively rare [9].

Lastly, MIGS can have a positive impact on patients’ quality of life. By achieving IOP control and reducing the need for multiple glaucoma medications, MIGS can alleviate the burden of complex medication regimens and their associated side effects, leading to improved treatment adherence and patient satisfaction [99,100,101].

## 5. Complications

As MIGS has become an increasingly popular treatment option, particularly for patients with mild-to-moderate OAG, it is essential to discuss potential complications associated with these procedures. Although MIGS is generally considered to have a favorable safety profile compared to traditional glaucoma surgeries, complications can still occur [102]. This section will explore the various complications that may arise following MIGS, their prevalence, and management strategies to mitigate potential risks.

One of the most frequently encountered complications following MIGS is transient hyphema, which is typically self-limited and resolves spontaneously within one to two weeks [1,2,6,103]. Patients may be advised to use a topical steroid to reduce inflammation and speed up the resolution of the hyphema. In rare cases, persistent or recurrent hyphema may require additional intervention, such as anterior chamber washout.

Another common complication is an early postoperative IOP spike, which may occur due to inflammation, retained viscoelastic material, or device obstruction [102,103,104]. These IOP spikes are generally transient and can be managed with topical IOP-lowering medications or, in some cases, oral carbonic anhydrase inhibitors [9]. If the IOP spike persists or is severe, further surgical intervention may be necessary.

Device-related complications can vary depending on the specific MIGS procedure performed. These complications may include malposition, migration, or obstruction of the device [1,2,6,102]. In some cases, a malpositioned or migrated device may require repositioning or removal, while device obstruction may be managed by flushing the device or administering IOP-lowering medications.

Although less common, serious complications can also occur following MIGS. Hypotony, characterized by a significant reduction in IOP, may lead to choroidal detachment, maculopathy, or optic disc swelling [103,104]. Management of hypotony may involve observation, adjustment of anti-inflammatory medications, or, in severe cases, surgical intervention to address the underlying cause. Infections, such as endophthalmitis, are rare but potentially vision-threatening complications following MIGS [9,105]. Prompt diagnosis and treatment with intravitreal antibiotics and possible surgical intervention are crucial to prevent permanent vision loss. Suprachoroidal hemorrhage is another rare complication that may result from excessive intraoperative bleeding [106]. In most cases, the hemorrhage is self-limiting and can be managed conservatively. However, in cases of significant or persistent bleeding, surgical intervention may be required [9].

While MIGS has a relatively favorable safety profile compared to traditional glaucoma surgeries, complications can still occur [107]. Clinicians must be aware of the potential complications and their appropriate management strategies to ensure optimal patient outcomes. Moreover, patient selection and proper surgical technique play vital roles in minimizing the risk of complications and maximizing the benefits of MIGS for patients with glaucoma.

## 6. Conclusions

MIGS has transformed the landscape of glaucoma management by providing a safer and less invasive alternative to traditional glaucoma surgeries. These innovative devices and procedures target various anatomical pathways to enhance aqueous humor outflow and effectively reduce IOP. The array of MIGS options, ranging from trabecular meshwork bypass stents to subconjunctival and suprachoroidal shunts, allows for tailored treatment strategies based on the specific needs and disease severity of each patient [108].

While MIGS has demonstrated promising results, it is essential to recognize that long-term outcomes and the sustainability of IOP control require further investigation. Comparative studies and randomized controlled trials will be crucial in establishing the relative efficacy and safety of MIGS devices and techniques compared to traditional glaucoma surgeries and medical management. Moreover, future research should focus on refining existing technologies and exploring new mechanisms of action to address the diverse etiologies of glaucoma and cater to the specific needs of patients who may not benefit from current MIGS options.

Ultimately, the growing field of MIGS signifies a paradigm shift in glaucoma management, offering hope for improved quality of life and visual outcomes for patients with this potentially blinding condition [109,110]. By continuing to expand our understanding of these devices, procedures, and surgical techniques, clinicians and researchers can further enhance patient care and strive towards the ultimate goal of preserving vision in those affected by glaucoma.

## Figures and Tables

**Table 1:** Summary of MIGS devices and techniques.

Device Category	Device(s) (Manufacturer(s))	FDA Approval Year	Brief Description	Mechanism of Action	Indication
Trabecular Outflow	Trabectome (NeoMedix Corporation)	2004	A microelectrocautery device that ablates and removes a portion of the trabecular meshwork and the inner wall of Schlemm’s canal.	Trabecular Meshwork Removal	Moderate-to-severe glaucoma
	Kahook Dual Blade (New World Medical)	2015	An instrument with a dual-sided tip for precise incisions to create a trabecular meshwork window.	Trabecular Meshwork Removal	Moderate-to-severe glaucoma
	iStent (Glaukos Corporation)	2012	The first trabecular micro-bypass stent implanted to enhance aqueous humor outflow.	Trabecular Bypass	Mild-to-moderate OAG
	iStent Inject (Glaukos Corporation)	2018	A preloaded injector system that implants two stents to enhance IOP reduction.	Trabecular Bypass	Mild-to-moderate OAG
	HFDS (Oertli Instrumente)	NA	Creates six pockets penetrating 1 mm deep into the trabecular meshwork and Schlemm’s canal.	Deep Sclerotomy	Moderate-to-severe OAG
Schlemm’s Canal Targeted	TRAB360 (Sight Sciences)	2013	A device utilizing a flexible microcatheter to cannulate and viscodilate Schlemm’s canal.	Trabecular Bypass	Mild-to-moderate OAG
	OMNI Surgical System (Sight Sciences)	2017	A two-step device that combines viscodilation of Schlemm’s canal and trabeculotomy to enhance aqueous humor outflow.	Viscodilation and Trabeculotomy	Mild-to-moderate OAG
	Streamline (New World Medical)	2021	A single-use device that creates a goniotomy and delivers viscoelastic material to dilate Schlemm’s canal.	Goniotomy and Canal Dilation	Mild-to-moderate OAG
	ABiC (Ellex iScience)	NA	Uses a microcatheter system to cannulate and dilate Schlemm’s canal and distal outflow channels with viscoelastic material.	Canal Dilation	Mild-to-moderate primary OAG
Schlemm’s Canal and Trabecular Outflow	Hydrus Microstent (Ivantis Inc.,)	2018	A flexible, biocompatible nitinol stent implanted via a clear corneal incision.	Canal Dilation and Trabecular Bypass	Mild-to-moderate OAG
Gonioscopy-Assisted Procedures	GATT	Not Device-Dependent	A procedure that utilizes a microcatheter and flexible suture for a 360-degree trabeculotomy.	360-degree Trabeculotomy	Moderate-to-severe glaucoma
Suprachoroidal Outflow	iStent Supra (Glaukos Corporation)	NA	A PES device with a porous titanium coating implanted to facilitate aqueous humor drainage into the suprachoroidal space.	Suprachoroidal Bypass	Moderate-to-severe glaucoma
Subconjunctival Shunts	EX-PRESS Glaucoma Filtration Device (Alcon Laboratories)	2002	Small, stainless-steel shunt that is available in two sizes: P-50 (50 microns) and P-200 (200 microns).	Bypasses the Trabecular Meshwork	Moderate-to-severe glaucoma
	Xen Gel Stent (Allergan)	2016	Hydrophilic, porcine-derived gelatin material, implanted to create a new drainage pathway that bypasses the trabecular meshwork.	New Drainage Pathway	Moderate-to-severe glaucoma
	PreserFlo MicroShunt (Santen)	2021	A small, flexible SIBS tube diverting aqueous humor from the anterior chamber to the subconjunctival space.	Diverts Aqueous Humor	Moderate-to-severe glaucoma

ABiC = Ab Interno Canaloplasty; GATT = gonioscopy-assisted transluminal trabeculotomy; HDFS = high-frequency deep sclerotomy; OAG = open-angle glaucoma; PES = polyethersulfone; NA = not applicable; SIBS = poly(styrene-block-isobutylene-block-styrene).

## Data Availability

No new data were created or analyzed in this study. Data sharing is not applicable to this article.
